# Case-Matched Outcomes of Proton Beam and Intensity-Modulated Radiation Therapy for Localized Prostate Cancer

**DOI:** 10.14338/IJPT-23-00002.1

**Published:** 2023-05-18

**Authors:** Alicia Bao, Andrew R. Barsky, Stefan Both, John P. Christodouleas, Curtiland Deville, Zelig A. Tochner, Neha Vapiwala, Russell Maxwell

**Affiliations:** 1 Ohio State College of Medicine, The Ohio State University, Columbus, OH, USA; 2 Department of Radiation Oncology, Lynn Cancer Institute, Baptist Health South Florida, Boca Raton, FL, USA; 3 Department of Radiation Oncology, University Medical Center Groningen, Groningen, the Netherlands; 4 Department of Radiation Oncology, University of Pennsylvania, Philadelphia, PA, USA; 5 Department of Radiation Oncology and Molecular Radiation Sciences, Johns Hopkins University, Baltimore, MD, USA

**Keywords:** proton therapy, radiation therapy, intensity-modulated radiation therapy, prostate cancer, secondary malignancy

## Abstract

**Purpose:**

Although both intensity-modulated radiation therapy (IMRT) and proton beam therapy (PBT) offer effective long-term disease control for localized prostate cancer (PCa), there are limited data directly comparing the 2 modalities.

**Methods:**

The data from 334 patients treated with conventionally fractionated (79.2 GyRBE in 44 fractions) PBT or IMRT were retrospectively analyzed. Propensity score matching was used to balance factors associated with biochemical failure-free survival (BFFS). Age, race, and comorbidities (not BFFS associates) remained imbalanced after matching. Univariable and covariate-adjusted multivariable (MVA) Cox regression models were used to determine if modality affected BFFS.

**Results:**

Of 334 patients, 176 (52.7%) were included in the matched cohort with exact matching to National Comprehensive Cancer Network (NCCN) risk group. With a median follow-up time of 9.0 years (interquartile range [IQR]: 7.8-10.2 years), long-term BFFS was similar between the IMRT and PBT matched arms with 8-year estimates of 85% (95% CI: 76%-91%) and 91% (95% CI: 82%-96%, *P* = .39), respectively. On MVA, modality was not significantly associated with BFFS in both the unmatched (hazard ratio [HR] = 0.75, 95% CI: 0.35-1.63, *P* = .47) and matched (HR = 0.87, 95% CI: 0.33-2.33, *P* = .78) cohorts. Prostate cancer–specific survival (PCSS) and overall survival (OS) were also similar (*P* > .05). However, in an unmatched analysis, the PBT arm had significantly fewer incidences of secondary cancers within the irradiated field (0.6%, 95% CI: 0.0%-3.1% versus 4.5%, 95% CI: 1.8%-9.0%, *P* = .028).

**Conclusions:**

Both PBT and IMRT offer excellent long-term disease control for PCa, with no significant differences between the 2 modalities in BFFS, PCSS, and OS in matched patients. In the unmatched cohort, fewer incidences of secondary malignancy were noted in the PBT group; however, owing to overall low incidence of secondary cancer and imbalanced patient characteristics between the 2 groups, these data are strictly hypothesis generating and require further investigation.

## Introduction

Despite fluctuations in screening guidelines, prostate cancer (PCa) is the most commonly diagnosed noncutaneous, solid malignancy in US men. As a broad range of management options exist for clinically localized PCa, from active surveillance to curative-intent treatment, patients obtain the most benefit from shared decision-making. Choosing definitive treatment such as surgery or radiation therapy (RT) is a multifaceted consideration incorporating life expectancy, clinical risk factors, and personal values regarding acceptable treatment-related toxicities. Furthermore, although active surveillance is the preferred option for patients with lower-risk disease, some patients may opt for definitive treatment owing to discomfort with delaying cancer care. For those who do choose to pursue RT, there are subsequent decisions to be made, including but not limited to technique (external beam radiation therapy [EBRT], brachytherapy, or combination), modality, dose, fractionation, and androgen deprivation therapy (ADT) use.

Both photon- and proton-based approaches are considered acceptable EBRT options for PCa. The most widely used photon-based technique in the United States is intensity-modulated radiation therapy (IMRT), preferred over 3-dimensional conformal radiation therapy (3D-CRT) because it typically permits safer dose escalation with a greater ability to spare surrounding organs at risk, thereby lowering long-term risks of gastrointestinal (GI) and genitourinary toxicities [1]. Proton beam therapy (PBT) is less frequently used than IMRT, but anticipated to increase with the opening of new proton centers across the United States. Given PBT’s physical properties and dosimetric advantages of reduced exit and integral dose, it is an appealing method for decreasing the overall volume of normal tissue irradiation, thus potentially lowering side effects and improving the therapeutic ratio. Previous dosimetric comparison studies have shown PBT compared favorably to IMRT, with significantly decreased low- and mid-dose radiation reaching nearby dose-limiting organs (eg, bladder and rectum), while delivering prescribed doses to the prostate target volume [2, 3]. Radiobiologic and epidemiologic studies have suggested that PBT is associated with decreased rates of secondary malignancy, a consideration that may be particularly appealing for younger patients receiving RT [4, 5]. In regard to clinical efficacy, a growing body of evidence demonstrates excellent disease-control outcomes with PBT [6–8]; however, these data are typically limited to single-arm PBT studies, with no direct comparison to patients treated with IMRT. Randomized controlled trial (RCT) data are the “gold standard” and eagerly awaited, but patients often inquire about evidence for the decisions they need to make today. Thus, the present study sought to conduct a propensity score–matched comparison of long-term outcomes between PBT and IMRT in clinically localized PCa.

## Materials and Methods

### Patient Selection

Patients with clinically localized PCa who underwent definitive PBT or IMRT at the Hospital of University of Pennsylvania between 2010 and 2012 were enrolled on institutional review board–approved prospective protocols evaluating outcomes of conventionally fractionated (CF) (the standard at the time) PBT or IMRT. All patients had histologically confirmed prostate adenocarcinoma, and any patients with metastatic disease or pelvic lymph node (LN) involvement at diagnosis were excluded. The decision between PBT and IMRT treatment was dependent on multiple factors including clinical suitability for PBT (anatomic and oncologic considerations), patient preference, treating physician preference, machine availability, and insurance coverage.

### Treatment Delivery

Radiation therapy treatment planning and delivery have been previously described in detail.^2^ The clinical target volume was designed as the entire prostate plus 1 cm of the proximal seminal vesicles. A 0.5-cm margin expansion was added in all directions for the planning target volume. All patients in this analysis, regardless of RT modality, were treated with CF (79.2 Gy relative biological effectiveness [RBE] in 44 fractions).

### Clinical Assessment

Electronic medical records were reviewed to collect baseline characteristics, including demographic data, clinical factors, and outcomes. Patients were stratified into their respective National Comprehensive Cancer Network (NCCN) risk group by their clinical stage, pretreatment serum prostate-specific antigen (PSA) level, and Gleason score from diagnostic prostate needle biopsy. Posttreatment serial PSA levels were monitored with biochemical failure (BF) defined by using Phoenix criteria (ie, rise of 2 ng/mL or greater above the post-RT nadir) [9]. If BF occurred and/or there were worrisome symptoms at clinic follow-up, further diagnostic studies (abdominopelvic imaging, nuclear bone scan, and/or prostate biopsy) were pursued to assess potential sites of biochemical recurrence. Local failure was defined as recurrence within the prostate or seminal vesicles, as detected by magnetic resonance imaging and/or confirmed by prostate biopsy. Regional failure was defined as metastases to the pelvic LNs. All other extrapelvic metastases were described as distant failure. Secondary, potentially RT-associated cancer was defined in 2 ways: as biopsy-proven, non–prostate malignancy with a latency of at least 5 years and (1) within the RT treatment field (low to high isodose lines, defined as the dose cloud bounded from the 1-Gy isodose line to the plan’s global hotspot) or (2) within the true pelvis (eg, pelvic colon, rectum, bladder, reproductive organs). Secondary malignancies within the true pelvis, irrespective of dose bath, were considered to address the potential bias that the IMRT dose bath inherently created a much larger volume of at-risk tissue.

### Study Design and Case Matching

The primary endpoint for this study was BFFS, with the main objective to compare this outcome between PBT and IMRT. Secondary endpoints included prostate cancer–specific survival (PCSS), overall survival (OS), patterns of failure, and secondary cancer rates. Toxicity outcomes for this cohort were not reevaluated, as they have been previously documented [2].

Given this is a single center, retrospective analysis, there are inherent limitations and selection biases (both measured and unmeasured). Thus, propensity score matching was used to case-match PBT and IMRT patients to better balance the treatment arms in regard to confounding variables that could influence the primary endpoint and study objective. These key variables were predetermined to be NCCN risk group (composite of clinical stage, serum PSA level, and pathologic Gleason score) and ADT use, as they are established predictors of BFFS. Age was a predetermined but secondary match variable, as younger patients may be more likely to be offered PBT for potential secondary malignancy risk concerns [10]. Using the MatchIt algorithm in R computing software (R Foundation for Statistical Computing, Vienna, Austria), patients in the PBT and IMRT treatment arms were matched by using exact matching for risk group (categorical variable with 4 levels) and nearest-neighbor matching for age (continuous variable) and ADT use (dichotomous variable). Although there is correlation between ADT use and risk group, investigators thought it would be important to include this variable in the matching algorithm because patients may not undergo ADT despite medical recommendation (eg, patient refusal, medical contraindication). Radiation therapy dose is known to significantly affect BFFS [11] but did not require matching, as total dose and fractionation were consistent across study patients. As a note, secondary cancer risk analyses were conducted by using the unmatched cohort because (1) matched patients were selected to adjust for potential confounders associated with BFFS, not secondary malignancy development; and (2) improvement of statistical power and detectability, as secondary cancers have very low incidences following RT.

### Statistical Analysis

All statistical analyses were carried out with SAS software, version 9.4 (SAS Institute, Cary, North Carolina). Baseline characteristics for both the unmatched and matched cohorts were compared between the PBT and IMRT treatment arms. Continuous variables were summarized by using medians, overall ranges, and interquartile ranges (IQRs), while categorical variables were summarized by using frequency counts and proportions. The nonparametric Mann-Whitney *U* and Fisher exact tests were used to compare continuous and categorical variables, respectively. All survival outcomes were calculated from RT treatment start. Patients with no survival event were censored from all survival analyses at their last oncologic clinic visit. Kaplan-Meier curves were constructed for all survival outcomes by using GraphPad Prism 9.3 (GraphPad Software Inc, San Diego, California) and compared by using log-rank tests. Univariable (UVA) and multivariable (MVA) Cox proportional hazard regression models were used to determine potential predictors of BFFS. Statistical tests were considered significant if associated 2-tailed *P* values were less than a predetermined type I error rate of .05.

## Results

### Baseline Characteristics in the Unmatched and Matched Cohorts

Patient baseline characteristics can be found in **Tables 1** and **2**. Three hundred thirty-four unmatched patients (**Table 1**) were initially evaluated, with 176 (52.7%) ultimately included in the matched cohort (**Table 2**). The overall unmatched cohort had significant imbalances between the PBT and IMRT treatment arms in regard to age, race/ethnicity, comorbidity, pretreatment PSA level, risk group, and ADT use. Once propensity score matched, there remained a significant difference in age, with the PBT arm being significantly younger (median age: 59 years, range: 59-84 years, IQR: 56-64 years) than the IMRT arm (median age: 67 years, range: 50-83 years, IQR: 62-71 years, *P* < .0001). This likely can be explained by the purposeful selection of younger patients as candidates for PBT given evidence of its association with decreased secondary malignancy risks [10]. There also remained a difference in race/ethnicity, with significantly more White patients comprising the PBT than the IMRT arm (74% versus 47%, *P* < .0001). Comorbidities including hypertension, hyperlipidemia, and diabetes mellitus varied significantly between the 2 arms even after matching, with significantly fewer people having comorbidities in the PBT arm (58% versus 76%, *P* = .016). This is likely in part related to the younger age of the PBT arm.

**Table 1.** Baseline patient characteristics for unmatched cohort (N = 334).

**Baseline characteristic**	**All - unmatched (N = 334)**	**IMRT (n = 157, 47%)**	**PBT (n = 177, 53%)**	***P*** **value^a^**
Age, y				**.0001**
Median [range, IQR]	66 [42-88, 60-70]	68 [50-88, 63-73]	64 [42-84, 59-69]	
Race/ethnicity, n (%)				**<.0001**
Non-White	112 (34)	79 (50)	33 (19)	
White	201 (60)	66 (42)	135 (76)	
Missing	21 (6)	12 (8)	9 (5)	
Comorbidity, n (%)				**<.0001**
No	106 (32)	33 (21)	73 (41)	
Yes	228 (68)	124 (79)	104 (59)	
Pre-RT PSA, ng/mL				**<0.0001**
Median [range, IQR]	5.2 [0.1-74.5, 4.2-7.5]	6.0 [0.1-40.9, 4.4-10.0]	4.9 [0.3-74.5, 3.9-6.5]	
Pre-RT PSA (categorical), n (%)				**<.0001**
<10.0 ng/mL	284 (85)	117 (75)	167 (94)	
>10.0–20.0 ng/mL	42 (13)	34 (22)	8 (5)	
>20.0–30.0 ng/mL	5 (2)	4 (3)	1 (<1)	
>30.0 ng/mL	3 (1)	2 (1)	1 (<1)	
NCCN risk group, n (%)				**<.0001**
LR	193 (58)	52 (33)	141 (80)	
FIR	59 (18)	34 (22)	25 (14)	
UIR	43 (13)	37 (24)	6 (3)	
HR	39 (12)	34 (22)	5 (3)	
ADT				**<.0001**
No	261 (78)	95 (61)	166 (94)	
Yes	73 (22)	62 (39)	11 (6)	
ADT duration, mo				.69
Median [range, IQR]	9 [4-39, 7-24]	9 [4-39, 7-24]	8 [5-36, 7-24]	
Follow-up time, y				**<.001**
Median [range, IQR]	8.8 [0.8-11.3, 7.7-9.8]	9.8 [0.8-11.3, 8.3-10.6]	8.3 [1.2-10.5, 7.6-9.0]	

**Abbreviations:** IMRT, intensity-modulated radiation therapy; PBT, proton beam therapy; IQR, interquartile range; RT, radiation therapy; PSA, prostate-specific antigen; NCCN, National Comprehensive Cancer Network; LR, low-risk; FIR, favorable intermediate-risk; UIR, unfavorable intermediate-risk; HR, high-risk; ADT, androgen deprivation therapy.

a Categorical and continuous variables were compared by using the nonparametric Fisher exact and Mann-Whitney *U* tests, respectively. Patients with missing values are shown for completeness but omitted from comparison statistical analyses.

**Table 2.** Baseline patient characteristics for matched cohort (N = 176).

**Baseline characteristic**	**All - matched (N = 176)**	**IMRT (n = 88, 50%)**	**PBT (n = 88, 50%)**	***P*** **value^a^**
**Age, y**				**<.0001**
Median [range, IQR]	63 [42-84, 58-70]	67 [50-83, 62-71]	59 [59-84, 56-64]	
Race/ethnicity, n (%)				**<.0001**
Non-White	62 (35)	42 (48)	20 (23)	
White	106 (60)	41 (47)	65 (74)	
Missing	8 (5)	5 (6)	3 (3)	
Comorbidity, n (%)				**.016**
No	58 (33)	21 (24)	37 (42)	
Yes	118 (67)	67 (76)	51 (58)	
Pre-RT PSA, ng/mL				.19
Median [range, IQR]	4.8 [0.1-74.5, 4.1-6.8]	4.9 [0.1-23.2, 4.2-7.3]	4.6 [0.7-74.5, 4.0-6.4]	
Pre-RT PSA, n (%)				.22
<10.0 ng/mL	159 (90)	77 (88)	82 (93)	
>10.0–20.0 ng/mL	14 (8)	10 (11)	4 (5)	
>20.0–30.0 ng/mL	2 (1)	1 (1)	1 (1)	
>30.0 ng/mL	1 (1)	0 (0)	1 (1)	
NCCN risk group, n (%)				1.00
LR	104 (59)	52 (59)	52 (59)	
FIR	50 (28)	25 (28)	25 (28)	
UIR	12 (7)	6 (7)	6 (7)	
HR	10 (6)	5 (6)	5 (6)	
ADT, n (%)				.66
No	151 (86)	74 (84)	77 (86)	
Yes	25 (14)	14 (16)	11 (13)	
ADT duration, mo				.70
Median [range, IQR]	8 [5-36, 7-19]	8 [6-36, 7-15]	8 [5-36, 7-24]	
Follow-up time, y				
Median [range, IQR]	9.0 [1.2-11.3, 7.8-10.2]	10.0 [1.4-11.3, 8.9-10.8]	8.4 [1.2-10.4, 7.5-9.1]	**<.0001**

**Abbreviations:** IMRT, intensity-modulated radiation therapy; PBT, proton beam therapy; IQR, interquartile range; RT, radiation therapy; PSA, prostate-specific antigen; NCCN, National Comprehensive Cancer Network; LR, low-risk; FIR, favorable intermediate-risk; UIR, unfavorable intermediate-risk; HR, high-risk; ADT, androgen deprivation therapy.

a Categorical and continuous variables were compared by using the nonparametric Fisher exact and Mann-Whitney *U* tests, respectively. Patients with missing values are shown for completeness but omitted from comparison statistical analyses.

Most importantly, risk group, ADT use, and pretreatment PSA levels were well balanced between the IMRT and PBT arms upon matching. Risk groups in both arms were exactly the same with primarily low-risk (LR) and favorable intermediate-risk (FIR) disease (87%), directly reflecting institutional protocol eligibility requirements during that period [12]. Median pretreatment PSA level was 4.9 ng/mL (range: 0.1-23.2 ng/mL, IQR: 4.2-7.3 ng/mL) and 4.6 ng/mL (range: 0.7-74.5 ng/mL, IQR: 4.0-6.4 ng/mL) in the matched IMRT and PBT groups, respectively (*P* = .19). Fourteen percent of patients received ADT with a median length of 8 months (range: 5-36 months, IQR: 7-19 months).

### Biochemical Failure-Free Survival

**Figure 1.**
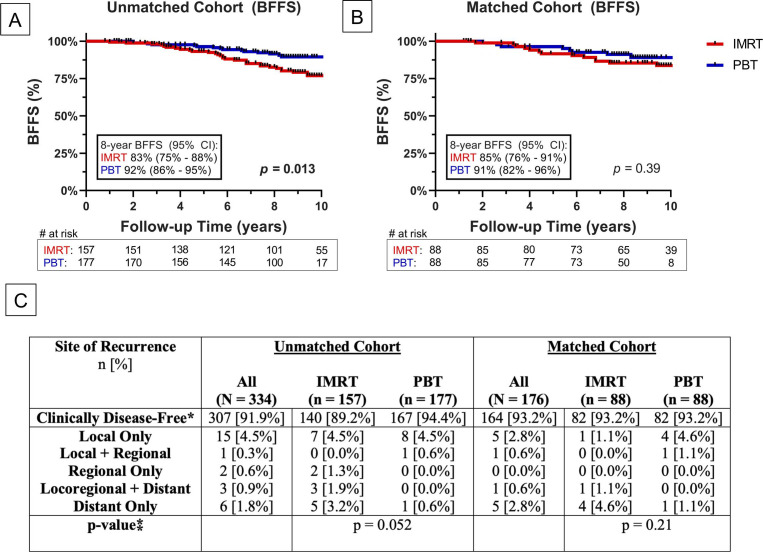
Long-term BFFS between IMRT (red) and PBT (blue) in the unmatched (A) and matched (B) cohorts. (C) Patterns of failure compared amongst IMRT versus PBT. *Clinically disease-free represents patients with no clinically observable sites of recurrence on follow-up physical examination, imaging, and/or biopsy. This does not necessary equate to biochemical recurrence. Abbreviations: BFFS, biochemical failure-free survival; CI, confidence interval; IMRT, intensity-modulated radiation therapy; PBT, proton beam therapy.

The median length of follow-up for the entire cohort was 9.0 years (range: 0.8-11.3 years, IQR: 7.8-9.8 years). In the unmatched cohort, BFFS was found to be significantly lower in the IMRT (8-year: 83%, 95% CI: 75%-88%) versus PBT (8-year: 92%, 95% CI: 86%-95%) arms (*P* = .013; **Figure 1A**), but this difference was no longer significant upon matching (*P* = .39; **Figure 1B**). In the matched cohort, 8-year BFFS was 85% (95% CI: 76%-91%) in the IMRT arm versus 91% (95% CI: 82%-96%) in the PBT arm (**Figure 1B**).

The UVA and MVA Cox regression models of BFFS can be found in **Table 3**. On UVA, RT modality was significantly associated with BFFS in the unmatched cohort, with the PBT arm having significantly improved BFFS (hazard ratio [HR] = 0.46, 95% CI: 0.25-0.87, *P* = .016). However, after adjusting for confounding variables, RT modality was no longer significant on MVA in the unmatched cohort (PBT versus IMRT, HR = 0.75, 95% CI: 0.35-1.63, *P* = .47). In concordance with these results, RT modality in the matched cohort remained an insignificant factor on both UVA (PBT versus IMRT, HR = 0.68, 95% CI: 0.28-1.64, *P* = .39) and MVA (PBT versus IMRT, HR = 0.87, 95% CI: 0.33-2.33, *P* = .78). The only other significant predictor of BFFS was risk group. Compared to LR patients, unfavorable intermediate-risk (UIR) patients had significantly worsened BFFS on UVA and MVA in both the matched and unmatched cohorts. Although high-risk patients had a trend towards worsened BFFS, there were not enough patient numbers and statistical power to show significance. Pretreatment PSA and ADT use were not significantly associated with BFFS in these study populations, nor were non–disease-related factors such as age, race, and comorbidity.

**Table 3.** UVA and MVA Cox regression analyses investigating predictors of BFFS.

**BFFS**	**Unmatched Cohort**		**Matched Cohort**	
	**UVA**	**MVA**	**UVA**	**MVA**
Variable	**HR [95% CI,** ***P*** **value]**	**HR [95% CI,** ***P*** **value]**	**HR [95% CI,** ***P*** **value]**	**HR [95% CI,** ***P*** **value]**
Age				
Units = 10 y	1.13 [0.76-1.63, .58]	0.97 [0.66-1.41, .86]	1.37 [0.82-2.29, .23]	1.16 [0.65-2.01, .62]
Race/ethnicity				
White	Reference	Reference	Reference	Reference
Non-White	1.64 [0.57-4.74, .36]	1.25 [0.41-3.78, .70]	0.97 [0.13-7.50, .98]	0.31 [0.03-3.16, .32]
Comorbidity				
No	reference	Reference	Reference	Reference
Yes	1.74 [0.86-3.51, .12]	1.42 [0.67-3.01, .36]	1.87 [0.69-5.08, .22]	2.67 [0.87-8.15, .085]
Pre-RT PSA				
Units = 10 ng/mL	1.30 [0.99-1.69, .057]	1.35 [0.91-2.00, .14]	1.12 [0.68-1.85, .66]	1.13 [0.24-5.22, .87]
NCCN risk group				
LR	Reference	Reference	Reference	Reference
FIR	**2.34 [1.11-4.96, .026]**	2.11 [0.94-4.72, .071]	1.90 [0.73-4.93, .19]	1.91 [0.70-5.20, .21]
UIR	**4.68 [2.24-9.78, <.0001]**	**3.71 [1.47-9.37, .0054]**	**6.40 [2.10-19.47, .0011]**	**10.20 [2.19-47.45, .0031]**
High-risk	2.04 [0.75-5.57, .16]	1.28 [0.28-5.93, .75]	NE [NE, .99]	NE [NE, .99]
ADT				
No	Reference	Reference	Reference	Reference
Yes	1.79 [0.94-3.40, .077]	0.94 [0.39-2.27, .90]	0.83 [0.19-3.54, .80]	0.41 [0.08-2.13, .29]
RT modality				
IMRT	Reference	Reference	Reference	Reference
PBT	**0.46 [0.25-0.87, .016]**	0.75 [0.35-1.63, .47]	0.68 [0.28-1.64, .39]	0.87 [0.33-2.33, .78]

**Abbreviations:** UVA, univariable analysis; MVA, multivariable analysis; BFFS, biochemical failure-free survival; HR, hazard ratio; CI, confidence interval; RT, radiation therapy; PSA, prostate-specific antigen; NCCN, National Comprehensive Cancer Network; LR, low-risk; FIR, favorable intermediate-risk; UIR, unfavorable intermediate-risk; NE, nonestimable; ADT, androgen deprivation therapy; IMRT, intensity-modulated radiation therapy; PBT, proton beam therapy.

Notes: Patients with missing values included in regression models to preserve statistical power; however, associated hazard ratios for missing subgroups are not shown as they are not clinically meaningful.

### Patterns of Failure

Patterns of failure for both the unmatched and matched cohorts can be seen in **Figure 1C**. Although there were low absolute rates of failure overall, local only was the most dominant clinical failure site in both the unmatched (n = 15, 4.5%) and matched (n = 5, 2.8%) cohorts. There were lower rates of local regional (unmatched: n = 1, 0.3%; matched: n = 1, 0.6%) and regional only (unmatched: n = 2, 0.6%; matched: n = 0, 0.0%) recurrences. Synchronous local regional and distant recurrence rates were 0.9% (n = 3) and 0.6% (n = 1) in the unmatched and matched cohorts, respectively. Six unmatched (1.8%) and 5 matched (2.8%) patients had distant only recurrences following definitive EBRT for their initially clinically localized PCa. These patterns of failures did not vary significantly between IMRT and PBT in either the unmatched (*P* = .052) or matched (*P* = .21) cohorts.

### Prostate Cancer–Specific Survival and Overall Survival

**Figure 2.**
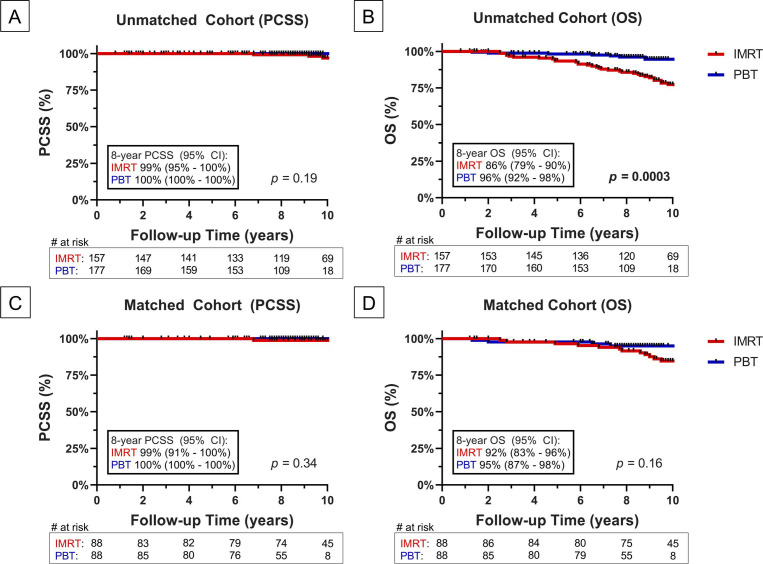
PCSS and OS between IMRT (red) and PBT (blue) in the unmatched (top panels, A and B) and matched (bottom panels, C and D) cohorts. Abbreviations: CI, confidence interval; IMRT, intensity-modulated radiation therapy; OS, overall survival; PBT, proton beam therapy; PCSS, prostate cancer–specific survival.

Forty patients died since the study concluded, with 3 deaths in the IMRT arm directly attributable to progressive PCa. Eight-year PCSS rates were 99% (95% CI: 95%-100%) and 100% (95% CI: 100%-100%) in the unmatched IMRT and PBT arms, respectively (*P* = .19; **Figure 2A**). These PCSS estimates did not vary significantly in the matched cohort (*P* = .34; **Figure 2C**). In the unmatched cohort, OS rates at 8 years were significantly lower in the IMRT (86%, 95% CI: 79%-90%) versus PBT arm (96%, 95% CI: 92%-98%, *P* = .0003; **Figure 2B**). This OS difference no longer held upon matching with 8-year OS rates being 92% (95% CI: 83%-96%) and 95% (95% CI: 87%-98%) in the IMRT and PBT arms, respectively (*P* = .16; **Figure 2D**).

### Secondary Cancer Rates

Among all patients (n = 334), 60 (18.0%, 95% CI: 14.0%-22.5%) were diagnosed with another nonprostate, primary cancer following RT, with no difference in rates between the IMRT and PBT arms (17.8%, 95% CI: 12.2%-24.7% versus 18.1%, 95% CI: 12.7%-24.6%, *P* = 1.00) (**Table 4**). In the unmatched cohort, the incidence of secondary cancer, defined as a biopsy-proven, non–prostate malignancy occurring more than 5 years after RT start within the RT field, was significantly lower in the PBT than IMRT arm (0.6%, 95% CI: 0.0%-3.1% versus 4.5%, 95% CI: 1.8%-9.0%, *P* = .028); however, when examining secondary cancers confined to the true pelvis, there were no statistically significant differences between the PBT and IMRT arms (0.6%, 95% CI: 0.0%-3.1% versus 3.2%, 1.0%-7.3%, *P* = .10). Matching was not performed for this portion of the study as the overall low incidence of secondary cancer development already limits statistical power. Specific secondary cancer diagnoses and latency periods can be found in **Table 4**. All nonprostate, primary cancer diagnoses following RT (in-field versus out-of-field) can be reviewed in **Supplemental Table S1**.

**Table 4.** Secondary cancer risks in unmatched cohort (N = 334).

**Cancer rates following RT**	**All (N = 334), n (%) [95% CI]**	**IMRT (n = 157), n (%) [95% CI]**	**PBT (n = 177), n (%) [95% CI]**	***P*** **value^a^**
Any cancer	60 (18.0) [14.0%-22.5%]	28 (17.8) [12.2%-24.7%]	32 (18.1) [12.7%-24.6%]	1.00
Secondary cancer^b^	8 (2.4) [1.0%-4.7%]	7 (4.5) [1.8%-9.0%]	1 (0.6) [0.0%-3.1%]	**.028**
Secondary cancer, pelvic^c^	6 (1.8) [0.7%-3.9%]	5 (3.2) [1.0%-7.3%]	1 (0.6) [0.0%-3.1%]	.10
Secondary cancer details Diagnosis [time from RT start, y]		(1) Rectal adenocarcinoma, mid-rectum [12.1] (2) Papillary urothelial carcinoma, high-grade, noninvasive, R distal ureter [5.5] (3) Osteosarcoma, R pubic ramus [11.3] (4) Papillary urothelial carcinoma, low-grade, noninvasive, anterior inferior bladder wall [5.3] (5) NHL (DLBCL), extranodal involvement with dominant L inguinal LN [6.7] (6) Metastatic bladder cancer, 2 primary bladder tumors involving anterior and R lateral dome [5.8] (7) Small cell carcinoma, prostatic urethra [11.1]	(1) Papillary urothelial carcinoma, low-grade, noninvasive, R posterolateral wall [7.5]	

**Abbreviations:** RT, radiation therapy; CI, confidence interval; IMRT, intensity-modulated radiation therapy; PBT, proton beam therapy; R/L, right/left laterality; NHL, Non-Hodgkin lymphoma; DLBCL, diffuse large B-cell lymphoma; LN, lymph node.

a Cancer rates were compared amongst IMRT versus PBT treatment arms by using the nonparametric Fisher exact test.

b Secondary, potentially RT-associated cancer defined as biopsy-proven, non–prostate malignancy within RT treatment field and latency period of at least 5 years from RT start.

c Refers to secondary cancers arising within the true pelvis (eg, pelvic colon, rectum, bladder, reproductive organs).

## Discussion

More comparative evidence is needed to establish outcome and toxicity differences in PBT versus IMRT for PCa. As we eagerly await results of the important prospective studies described below, we present the first ever case-matched study directly comparing long-term disease-control outcomes in patients who received CF PBT versus IMRT. Attributed to pretreatment imbalances in disease severity, there was a statistically significant difference in BFFS between IMRT and PBT in the unmatched cohort that was no longer significant upon propensity score matching, with 8-year BFFS estimates of 85% and 91% in the IMRT and PBT arms, respectively (*P* = .39). These results were in agreement in the MVA, with RT modality *not* being a significant factor contributing to BFFS in either the unmatched (PBT versus IMRT, HR = 0.75, *P* = .47) or matched (PBT versus IMRT, HR = 0.87, *P* = .78) cohorts. These data are largely expected, given both cohorts were treated to the same prescription dose, with the differential RBE of protons accounted for within the treatment planning process. Radiation therapy dose is a well-established predictive factor for biochemical control, and our 8-year BFFS rates are consistent with those reported in RTOG 0126 for the 79.2-Gy arm, acknowledging that our study population included lower-risk patients [13]. The present analysis provides reassurance of comparable long-term disease outcomes in the treatment of PCa when similar doses are prescribed.

The initial toxicity outcomes of this cohort have previously been described in another case-matched study using both qualitative physician-assessed and patient-reported symptoms. There was a reduction in acute GI toxicity reported in the PBT arm, but it was no longer significant after accounting for preexisting comorbidities [2]. On MVA, no significant differences were seen in acute or late grade 2+ genitourinary and GI toxicities between the PBT and IMRT arms.

In the present study, patients in the unmatched cohort treated with PBT were found to have lower rates of secondary malignancies within the RT field and within the true pelvis, although the latter observation was not statistically significant. These findings are concordant with some radiobiological modeling and database reports; however, long-term clinical data are limited. Specific to PCa, a risk model performed by Yoon et al [14] demonstrated that IMRT resulted in higher incremental radiation doses to normal tissue on an order of magnitude greater than PBT, leading to the prediction that IMRT would increase secondary cancer risks by 5-fold [14]. A recent National Cancer Database analysis was consistent with these modeling reports and found PCa patients undergoing PBT were at least 4 times less likely to have a subsequent secondary malignancy than with IMRT [4]. The documented benefit of PBT is the reduction of integral dose, with IMRT exposing larger volumes of surrounding normal tissue to low RT doses (ie, “low-dose bath”). Secondary RT-induced cancer risk models postulate the volume exposed to low doses are possibly more prone to cancer development because sterilization of mutated cells becomes more dominant at higher doses [15, 16]. Stray neutron contamination is a concern with PBT, but data from Monte Carlo simulations still demonstrate lower secondary cancers with PBT versus IMRT, taking into account this secondary radiation [17]. Moreover, newer PBT techniques such as pencil-beam scanning have lower neutron contamination than older techniques.

Nevertheless, there are important caveats with interpreting the secondary cancer findings, including the differential follow-up time (unmatched: IMRT versus PBT, 9.8 versus 8.3 years, Δ1.5 years, *P* < .001), imbalanced patient characteristics in the unmatched cohort, unmeasured factors biasing the results, and inability to prove causation with radiation. The latter points are important to consider, as unmatched IMRT patients could inherently have higher cancer predispositions, which were not adjusted for in the analysis and influenced the results rather than RT modality. For instance, age is known to be a risk factor for cancer development, and IMRT patients were older than PBT patients by approximately 4 years in the unmatched cohort. Although provocative data, these secondary malignancy findings are not definitive and must be corroborated with other comparison studies that include larger cohorts and longer follow-up to better estimate secondary cancer risk differences between IMRT and PBT in PCa.

Inherent to any single center, retrospective experience, the present study has limitations related to its primary endpoint, including potential selection biases that could confound the results. Propensity score matching was used to balance the significant differences in baseline characteristics between the treatment arms to better account for confounding and to reduce the selection bias associated with treatment assignment. The propensity score matching algorithm caused a significant reduction of patient numbers with 158 of the initial 334 patients (47.3%) being excluded from the final matching study. After matching, there remained lingering imbalances in (age, race, and comorbidities), indicating distinct differences between the 2 cohorts; however, these imbalanced factors are generally not associated with BFFS, which was considered the primary endpoint. More importantly, the matched cohorts were well balanced according to factors known to affect BFFS (risk group, pretreatment PSA level, and ADT use). Radiation therapy dose did not have to be adjusted for because all patients were conveniently prescribed the same dose of 79.2 GyRBE in 44 fractions. Although subjects enrolled on this prospective protocol received CF, the standard when the study started 12 years ago, more hypofractionated (HF) schemes are the new standard and would likely yield similar results from modality comparisons, given comparable efficacy of HF and CF established in numerous RCTs using IMRT/3D-CRT [18, 19], and similar nonrandomized data supporting HF PBT in PCa [20].

In practice, the age imbalance was expected as PBT has been favored in younger patients owing to reduced association with secondary malignancy [10]. In contrast, the marked racial imbalance was disconcerting and further highlights deep-seated health disparities and inequities seen in access to proton therapy, oncologic care, and US health care in general. These findings are in line with a prior study demonstrating that significantly fewer Black or African American patients received PBT for PCa relative to their White counterparts [21]. Nonetheless, these data provide important insights into persistent health care disparities within radiation oncology care, especially with respect to newer technologies. To the extent that any therapeutic advance is clinically appropriate and indicated for a given situation, sociodemographic characteristics such as race/ethnicity and socioeconomic status should not pose a barrier, especially as availability of said technology, and in turn affordability, increase. These data should serve as further motivation for institutions and for the field to enact policies that directly address factors contributing to unequal access, including structural and systemic inequities and implicit and explicit biases.

Despite its limitations, the present study should provide some reassurance while we await higher-level evidence from ongoing RCTs. The PARTIQoL trial (NCT01617161) is an ongoing phase III RCT with the primary goal to compare long-term bowel toxicity differences at 2 years between PBT and IMRT in LR and FIR PCa [22]. Additionally, the COMPPARE trial (NCT03561220) is studying quality-of-life and disease control in parallel cohorts of men treated with PBT and IMRT, with subgroup analyses focusing on fractionation schedule [23].

## Conclusion

This study represents the first propensity score–matched studies comparing long-term biochemical control outcomes between IMRT and PBT in clinically localized PCa and offers a direct comparison between the 2 treatment modalities. Both RT modalities were found to offer excellent and comparable long-term disease-control outcomes. The data showing reduced secondary malignancy rates in the PBT group are strictly hypothesis generating and need further investigation. In the context of previously reported toxicity studies, PBT appears to be a reasonable and safe option for clinically localized PCa. Prospective randomized trials are pending to further elucidate modality-dependent differences in short- and long-term outcomes after definitive-intent IMRT and PBT for clinically localized PCa.
