# Supplementary material for: Case-Matched Outcomes of Proton Beam and Intensity-Modulated Radiation Therapy for Localized Prostate Cancer
Source: Int J Part Ther. 2023 May 18;10(1):1–12. doi: 10.14338/IJPT-23-00002.1 (PMC10563661; doi:10.14338/IJPT-23-00002.1)
Supplement: Supplementary file 1 [file ijpt-10-01-04_s01.docx]

**Supplementary Table S1|** List of all non-prostate cancer diagnoses following RT in unmatched cohort (N = 334)

| **Cancer diagnoses**  **following RT** [time from RT start, years] | |
| --- | --- |
| **IMRT (n = 157)** | |
| **Out-of-field**  [total 19 events] | **In-field**  [total 9 events] |
| **(1)** Renal cell carcinoma (clear cell) [11.6]  **(2)** Metastatic colon adenocarcinoma, transverse colon [8.7]  **(3)** Early-stage NSCLC (adenocarcinoma), LLL [6.7]  **(4)** Pancreatic adenocarcinoma [10.5]  **(5)** Non-melanoma skin cancer (BCC), H&N [1.1]  **(6)** Multiple myeloma [9.2]  **(7)** Renal cell carcinoma (clear cell) [11.6]  **(8)** Non-melanoma skin cancer (BCC & SCC), H&N [9.6]  **(9)** Early-stage NSCLC (NOS), LUL [6.5]  **(10)** Melanoma, L UE [7.6]  **(11)** Oropharyngeal HNSCC (p16+), R tonsil [4.2]  **(12)** Renal cell carcinoma (NOS), multifocal [unknown]  **(13)** Acute myeloid leukemia [8.5]  **(14)** Metastatic NSCLC (adenocarcinoma), LUL [3.5]  **(15)** Early-stage NSCLC (adenocarcinoma), LUL [10.0]  **(16)** Renal cell carcinoma (clear cell) [2.5]  **(17)** Non-melanoma skin cancer (SCC), H&N [unknown]  **(18)** NHL (MZL), supra- and infradiaphragmatic disease [4.9]  **(19)** NHL (MZL), L eye [2.4] | **(1)** Rectal adenocarcinoma, mid-rectum [12.1]*†  **(2)** Papillary urothelial carcinoma, high-grade, non-invasive, R distal ureter [5.5]*†  **(3)** Osteosarcoma, R pubic ramus [11.3]†  **(4)** Papillary urothelial carcinoma, low-grade, non-invasive, anterior inferior bladder wall [5.3]*†  **(5)** Colon adenocarcinoma, sigmoid colon [3.6]*  **(6)** Papillary urothelial carcinoma, low-grade, non-invasive [4.2]*  **(7)** NHL (DLBCL), extranodal involvement with dominant L inguinal LN [6.7]†  **(8)** Metastatic bladder cancer, 2 primary bladder tumors involving anterior and R lateral dome [5.8]*†  **(9)** Small cell carcinoma, prostatic urethra [11.1]*† |
| **PBT (n = 177)** | |
| **Out-of-field**  [total 28 events] | **In-field**  [total 3 events] |
| **(1)** Soft tissue sarcoma, thoracolumbar spine [2.8]  **(2)** Metastatic NSCLC (SCC) [11.0]  **(3)** Non-melanoma skin cancer (SCC), R distal LE [7.4]  **(4)** Renal cell carcinoma (NOS) [10.0]  **(5)** Metastatic NSCLC (adenocarcinoma) [unknown]  **(6)** Melanoma, H&N [10.1]  **(7)** Early-stage NSCLC (adenocarcinoma) [7.1]  **(8)** Chronic lymphocytic leukemia, H&N [5.2]  **(9)** Follicular thyroid carcinoma [2.1]  **(10)** Metastatic NSCLC (NOS) [4.9]  **(11)** NHL (DLBCL), supra- and infradiaphragmatic [8.8]  **(12)** Melanoma, H&N [unknown]  **(13)** NHL (DLBCL), extranodal involvement of lumbar spine [6.6]  **(14)** Metastatic renal cell carcinoma (clear cell) [7.5]  **(15)** Melanoma, UE [unknown]  **(16)** Early-stage NSCLC (adenocarcinoma) [2.1]  **(17)** Non-melanoma skin cancer (SCC), chest [2.0]  **(18)** Hypopharyngeal HNSCC [5.9]  **(19)** Non-melanoma skin cancer (BCC), H&N [1.4]  **(20)** Melanoma [unknown]  **(21)** Pancreatic adenocarcinoma [8.0]  **(22)** Colon adenocarcinoma, cecum [9.3]  **(23)** Gastric adenocarcinoma [5.0]  **(24)** Melanoma & non-melanoma skin cancer (BCC) [unknown]  **(25)** Melanoma, H&N [5.0]  **(26)** Extrahepatic biliary adenocarcinoma [0.4]  **(27)** Non-melanoma skin cancer (BCC), H&N [4.3]  **(28)** Non-melanoma skin cancer (SCC), H&N [5.7] | **(1)** Papillary urothelial carcinoma, low-grade, non-invasive, R posterolateral wall [7.5] *†  **(2)** Papillary urothelial carcinoma, low-grade, non-invasive, posterior bladder dome [3.7] *  **(3)** Papillary urothelial carcinoma, high-grade, prostatic urethra [3.3]* |

Notes: *Refers to cancers arising within the true pelvis (pelvic colon, rectum, bladder, reproductive organs, etc). †Refers to a secondary, potentially RT-associated cancer, which was defined as a biopsy-proven, non-prostate malignancy within RT treatment field and latency period of at least 5 years from RT start. Abbreviations: RT, radiotherapy; IMRT, intensity-modulated radiotherapy (IMRT); PBT, proton beam therapy; R/L, right/left laterality; NSCLC, non-small cell lung cancer; LLL, left lower lobe; LUL, left upper lobe; BCC, basal cell carcinoma; SCC, squamous cell carcinoma; H&N, head and neck; NOS, not otherwise specified; UE, upper extremity; HNSCC, head and neck squamous cell carcinoma; NHL, Non-Hodgkin lymphoma; MZL, marginal zone lymphoma; DLBCL, diffuse large B-cell lymphoma; LN, lymph node.
